# Infectious Keratitis: A Retrospective Analysis of a Tertiary Care Center

**DOI:** 10.3390/jcm15114249

**Published:** 2026-05-30

**Authors:** Jana Schaetzel, Taos Batal, Marcus Walckling, Thomas A. Fuchsluger

**Affiliations:** Department of Ophthalmology, University Medical Center Rostock, 18057 Rostock, Germany; taos.batal@gmail.com (T.B.); marcus.walckling@med.uni-rostock.de (M.W.); thomas.fuchsluger@med.uni-rostock.de (T.A.F.)

**Keywords:** infectious keratitis, viral keratitis, bacterial keratitis, fungal keratitis, *Acanthamoeba* keratitis

## Abstract

**Background/Objectives:** Infectious keratitis is a vision-threatening disease. Its prevalence and specific pathogens vary by geographic location. This study characterizes risk profiles, clinical manifestations, and treatment outcomes for various pathogens in the Rostock area. **Results:** The study included 65 patients (38 viral, 14 bacterial, seven fungal, six parasitic) with a minimum follow-up of three months. The cohort had a mean age of 59 ± 19 years, with 49% female and 51% male participants. All groups showed significant improvement in visual acuity (viral −0.3 logMAR, *p* = 0.011; parasitic −0.8 logMAR, *p* = 0.043; fungal −0.9 logMAR, *p* = 0.018; Wilcoxon). Only the bacterial group did not reach statistical significance (−0.3 logMAR, *p* = 0.169; Wilcoxon). Final visual acuity did not differ significantly between medical and surgical treatments. **Conclusions:** No treatment modality (medical vs. surgical) showed superiority regarding visual outcome across pathogen groups. Early diagnosis and prompt therapy initiation are essential to improve visual prognosis and reduce complications.

## 1. Introduction

Infectious keratitis remains a leading cause of corneal opacity and severe visual impairment [1]. According to WHO estimates, approximately 1.5 million people worldwide go blind each year due to infectious corneal ulcerations, representing a significant global health burden [2]. The causative spectrum—comprising viruses, bacteria, parasites and fungi—exhibits substantial regional variation [3,4,5]. Risk factors include contact lens wear, ocular trauma, ocular surface disease (OSD), ocular surgery and systemic diseases such as diabetes mellitus [3,4,5,6,7].

**Figure 1:**
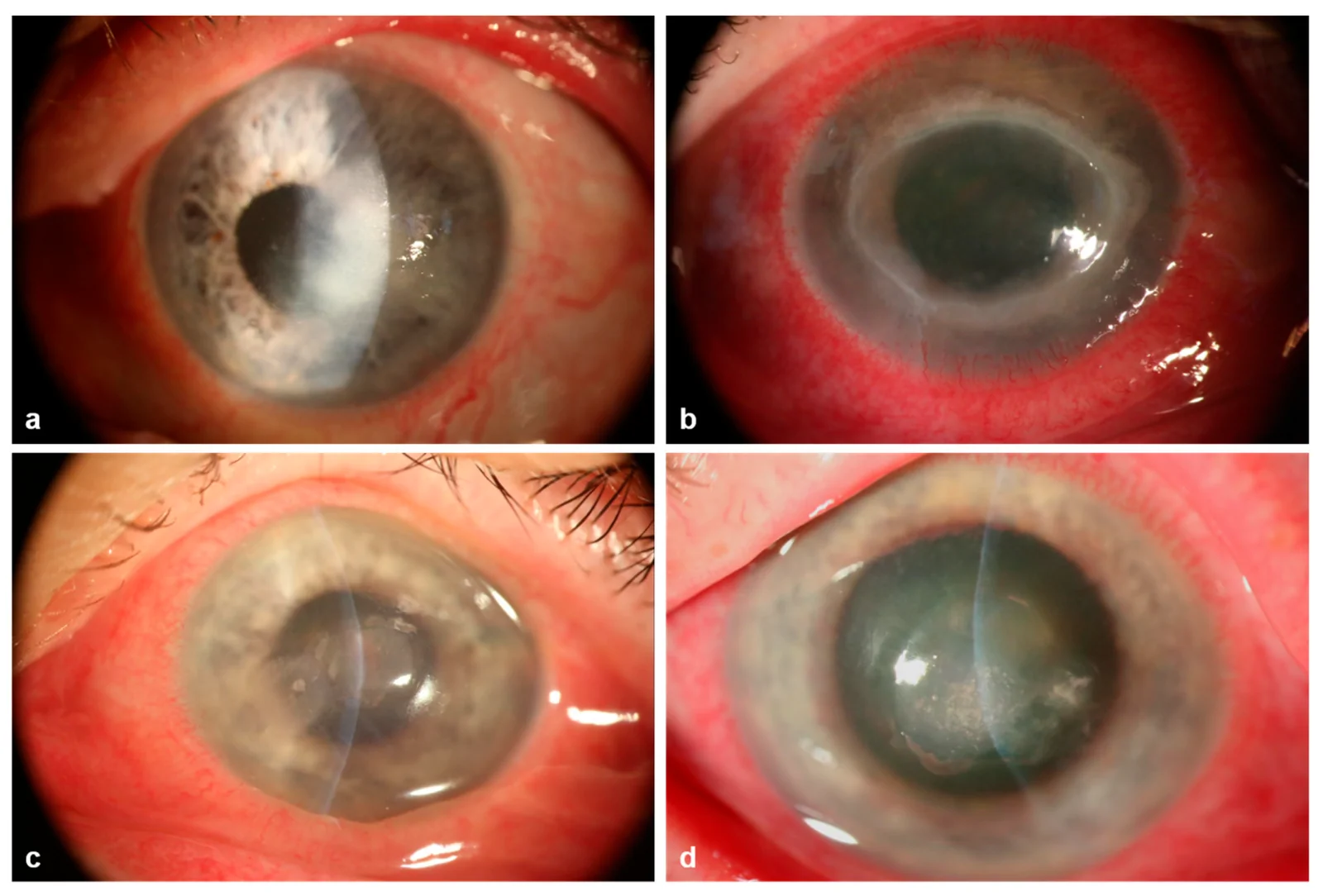
(**a**): Corneal infiltrate in a patient with viral keratitis. (**b**): Corneal infiltrate with ring-shaped configuration in a patient with *Acanthamoeba* keratitis. (**c**): Corneal infiltrate in a patient with bacterial keratitis. (**d**): Corneal infiltrate in a patient with fungal keratitis. All images were captured at the Department of Ophthalmology, University Medical Center Rostock.

Clinical findings may vary depending on the pathogen. Typical findings in viral keratitis are branching epithelial defects (“dendrites”) and reduced corneal sensitivity. *Acanthamoeba* keratitis is characterized by ring-shaped infiltrates. Bacterial corneal infections also show whitish-yellowish infiltrates and may be accompanied by hypopyon. Mycotic infections lead to raised infiltrates with feathery margins and possible satellite lesions [Figure 1]. While these clinical hallmarks are well-established, prompt and precise pathogen identification remains a challenge. Despite the availability of advanced molecular techniques like PCR or in vivo confocal microscopy (IVCM), the diagnostic yield in clinical practice varies [4,5,8,9].

Management of infectious keratitis has become increasingly complex due to shifting pathogen spectra and evolving resistance patterns. Standardized protocols ranging from intense monotherapy to combination regimens are essential to prevent progression. Treatment of viral keratitis consists of topical antiviral therapy. In cases of deep corneal infiltrates or intraocular involvement, systemic antiviral agents should be added and continued at low doses as a prophylactic measure against recurrence [10,11]. *Acanthamoeba* infections are treated with topical biguanides and diamidine. Due to frequent co-infections, topical antibiotics should be used as well [5,12]. If bacterial keratitis is suspected, empirical treatment with a broad-spectrum antibiotic is recommended. If the intraocular structures are affected, systemic antibiotic therapy is initiated [13]. Mycotic infections primarily require the use of topical antifungal agents such as azoles, polyene, and pyrimidine [14].

However, the use of adjunctive therapies such as steroids and the timing of surgical interventions such as penetrating keratoplasty remain subjects of ongoing debate [1,8,15].

Long-term data spanning several decades are scarce but crucial to understanding the clinical trajectory and interventional requirements of these patients. This study aimed to analyze a 20-year cohort of infectious keratitis in Northeast Germany to characterize risk factors, diagnostic yields and therapeutic outcomes, with a specific focus on the factors necessitating surgical intervention.

## 2. Materials and Methods

### 2.1. Study Design and Population

**Figure 2:**
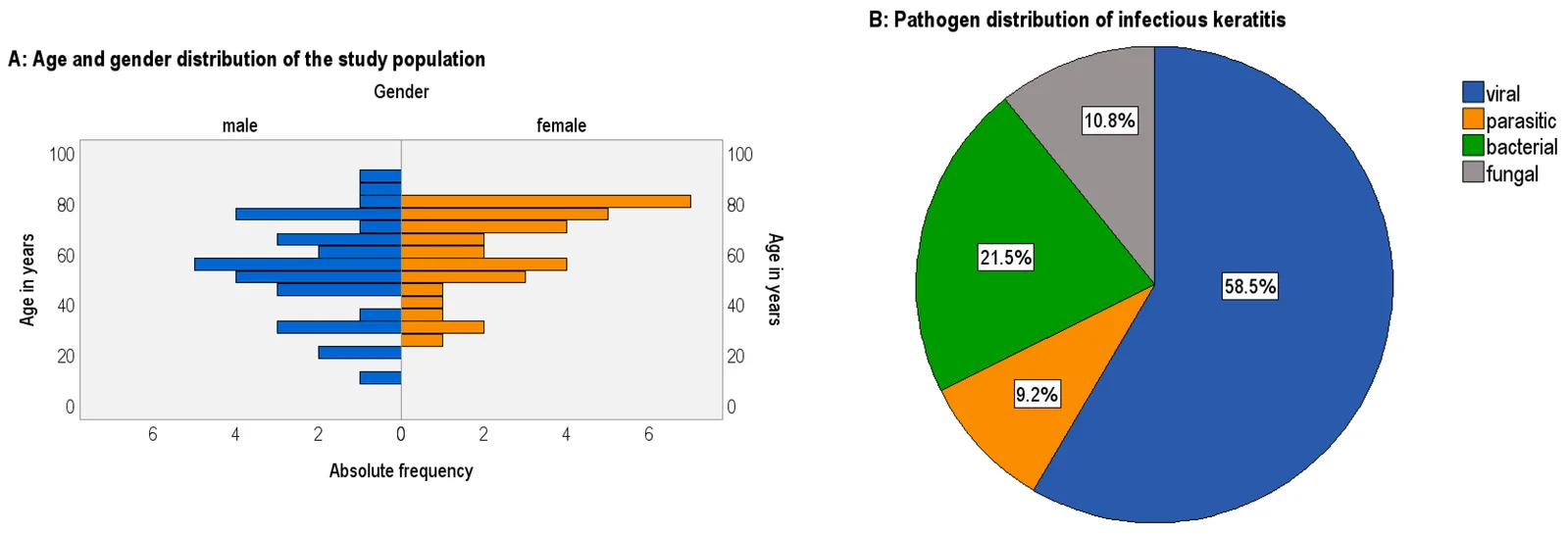
*n* (total) = 65, *n* (viral) = 38, *n* (parasitic) = 6, *n* (bacterial) = 14, *n* (fungal) = 7. (**A**): Age and gender distribution of the study population. The age and gender distribution was divided according to pathogen group: viruses, *Acanthamoeba*, bacteria and fungi. Age groups were considered separately for men and women. (**B**): Pathogen distribution of infectious keratitis: Viral, *Acanthamoeba*, bacterial, and fungal keratitis occurred.

Patients with infectious keratitis from 2000 to 2019 and a minimum follow-up period of 3 months were identified at the University Eye Clinic Rostock and the Medical Care Center for Ophthalmology Rostock. Pathogen detection data were obtained from the laboratory information system Swisslab in collaboration with the Institute for Medical Microbiology, Virology and Hygiene. A total of 65 patient records were reviewed retrospectively. Data regarding demographic, epidemiological and anamnestic parameters, as well as therapeutic outcomes, were collected. Severity was graded according to the “Manchester Keratitis Study” criteria by Efron and Morgan [16], supplemented by additional clinical findings [Table 1]. Patients with herpetic keratitis were included in the viral keratitis group [Figure 2]. Adenovirus infections were not considered, as they typically manifest as epidemic keratoconjunctivitis and do not usually require surgical interventions.

### 2.2. Diagnostic Pathway

Diagnostic procedures followed a standardized clinical protocol at our tertiary care facility. All patients underwent a slit-lamp examination upon admission. In cases of unclear clinical presentation, a stepwise diagnostic approach was employed. This included corneal swabs and scrapings for culture and PCR (addressing viral, *Acanthamoeba*, bacterial and fungal DNA). If anterior chamber involvement was present, an anterior chamber tap was performed. In vivo confocal microscopy was used as an adjunctive tool, depending on the availability of experienced examiners. The definitive diagnosis for this retrospective analysis was primarily based on positive laboratory results or characteristic IVCM findings. Overall, a positive organism or characteristic diagnostic marker was identified in 26/65 cases (40.0%). Pathogen detection rates varied by group: viral 13/38 (34.2%), *Acanthamoeba* 4/6 (66.7%, via IVCM), bacterial 7/14 (50.0%) and fungal 4/7 via culture and 1/7 via IVCM (total 71.4%). In the absence of pathogen detection, diagnosis relied on clinical presentation and clinical improvement under pathogen-specific therapy.

All patients were treated according to the specialist standard of care. Clinical examinations and therapeutic decisions were performed by or supervised by senior board-certified ophthalmologists to ensure a high quality of treatment.

### 2.3. Statistics

Statistical analysis was performed using IBM SPSS Statistics (Version 27). The Mann–Whitney U test was used for non-normally distributed independent samples, and the Wilcoxon test was used for non-normally distributed dependent samples. The chi-square and Fisher’s exact tests were used to analyze categorized frequencies statistically. The significance level was set at α = 0.05; results with *p* < 0.05 were considered statistically significant.

## 3. Results

### 3.1. Epidemiological Data and Spectrum of Pathogens

Of the 65 patients with infectious keratitis and a minimum three-month follow-up, pathogen distribution was as follows:

- Viral: 38 (58.5%).
- Bacterial: 14 (21.5%).
- Fungal: 7 (10.8%).
- Parasitic (*Acanthamoeba*): 6 (9.2%).

Gender distribution showed a descriptive trend. Women were more frequently affected by viral and *Acanthamoeba* keratitis, while men predominated the bacterial and fungal cases. However, this difference was not statistically significant (*p* = 0.140, Fisher’s exact test). Statistical analysis revealed no significant difference in age across the four pathogen groups, although a slight trend was observed (*p* = 0.069, Kruskal–Wallis test) [Table 2].

### 3.2. Risk Factors

The primary risk factor for *Acanthamoeba* keratitis was contact lens wear (83.3%). Specific exposures included wearing lenses in a swimming pool or using contaminated lenses (one case each). Additional risk factors for all pathogen groups can be found in Table 2.

### 3.3. Symptoms and Clinical Findings

Clinical findings varied by pathogen group [Table 2]. Among viral keratitis cases, combined infections and keratouveitis were most frequent (28.9% each), followed by interstitial (15.8%), epithelial (13.2%), necrotizing (10.5%), and endothelial keratitis (2.6%).

**Figure 3:**
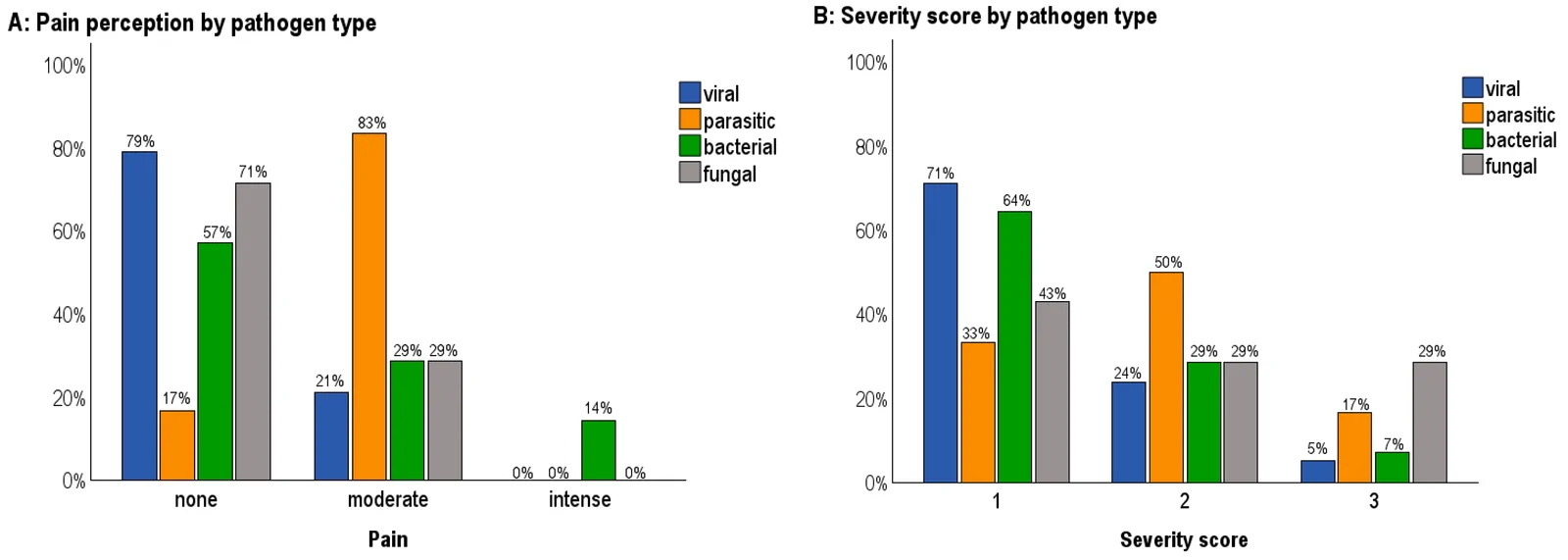
*n* (total) = 65, *n* (viral) = 38, *n* (parasitic) = 6, *n* (bacterial) = 14, *n* (fungal) = 7. (**A**): Pain perception by pathogen type: Pain perception was divided into “none”, “moderate” and “intense”. A statistically significant difference was observed regarding pain perception across the pathogen groups (*p* = 0.009, Fisher’s exact test). (**B**): Severity score by pathogen type: Infectious keratitis was divided into severity grades 1 = mild, 2 = moderate and 3 = severe using the classification system developed by Efron and Morgan in the Manchester Keratitis Study and the supplementary clinical findings in Table 2. No statistically significant difference in severity score was observed between the pathogen groups (*p* = 0.232, Fisher’s exact test).

Figure 3 presents the reported pain perception and severity scores. A statistically significant difference in pain perception was observed across the four cohorts (*p* = 0.009, Fisher’s exact test). Patients with *Acanthamoeba* keratitis reported disproportionally higher levels of pain.

### 3.4. Diagnostics

#### 3.4.1. Time Between Symptom Onset and First Consultation

**Figure 4:**
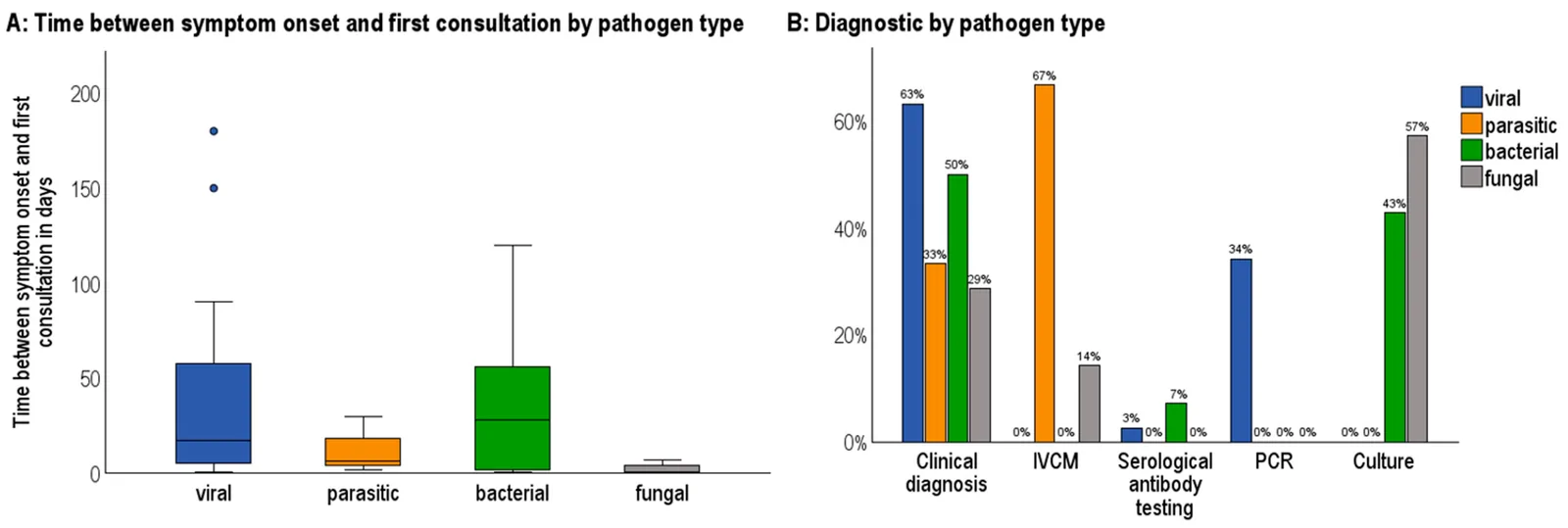
(**A**): Time between symptom onset and first consultation by pathogen group: The duration between the symptom onset and the first consultation in days is reported for each pathogen group. Blue circles indicate outliers. Data regarding symptom onset was available in 38 patients (viral: *n* = 20, parasitic: *n* = 4, bacterial: *n* = 9, fungal: *n* = 5). (**B**): Diagnostics by pathogen group: The methods leading to the correct diagnosis of infectious keratitis are listed. PCR = polymerase chain reaction. *n* (total) = 65, *n* (viral) = 38, *n* (parasitic) = 6, *n* (bacterial) = 14, *n* (fungal) = 7.

Time between symptom onset and first consultation varied widely [Figure 4A]:

- Viral: 40 (±50) days (median: 18 days).
- Bacterial: 35 (±41) days (median: 28 days).
- *Acanthamoeba*: 11 (±13) days (median: 7 days).
- Fungal: 3 (±3) days (median: 1 day).

Notably, viral keratitis patients with moderate-to-severe pain sought medical attention significantly earlier (median: 3 days) than those with mild-to-no pain (median: 30 days) (*p* = 0.017, Mann–Whitney U test). No significant correlations were found between timing of the consultation and patient age or severity score.

#### 3.4.2. Diagnostic Yield and Pathogen Identification

Viral: Pathogens were confirmed in 13/38 cases (34.2%). HSV-1 DNA was detected via PCR in 12 patients (31.6%) and one additional case was confirmed through serological antibody testing (primary infection). In PCR-negative cases, diagnosis was based on characteristic clinical findings: dendritic ulcers were present in eight patients (21.1%) and corneal hypoesthesia was documented in 22 patients (57.9%).

*Acanthamoeba*: Two cases were initially misdiagnosed, one with herpes keratitis, and one patient with an erosion after contact lens removal. Diagnosis was confirmed via in vivo confocal microscopy in four cases (66.7%), while two patients (33.3%) were diagnosed clinically.

Bacterial: Confirmation was achieved by culture-based methods in six cases (42.9%) and by PCR-based DNA detection in one case (7.1%). Identified species included *Staphylococcus aureus* (*n* = 2) followed by *Pseudomonas aeruginosa*, *Serratia marcescens*, *Enterococcus faecium*, *Lactobacillus* spp., and *Bacillus cereus* (*n* = 1 each). In the remaining seven cases (50.0%), where laboratory results were negative or unavailable, the diagnosis was established based on clinical presentation and response to therapy.

Fungal: Pathogen identification was successful in 5/7 patients (71.4%). Culture confirmed *Fusarium* spp. in four patients (57.1%), including co-infections with *Candida parapsilosis* and *Rhodotorula* spp. Confocal microscopy identified hyphae in one case (14.3%). The remaining two cases (28.6%) were diagnosed based on typical clinical findings and improvement under specific therapy [Figure 4B].

### 3.5. Management

#### 3.5.1. Medical and Surgical Therapy

**Figure 5:**
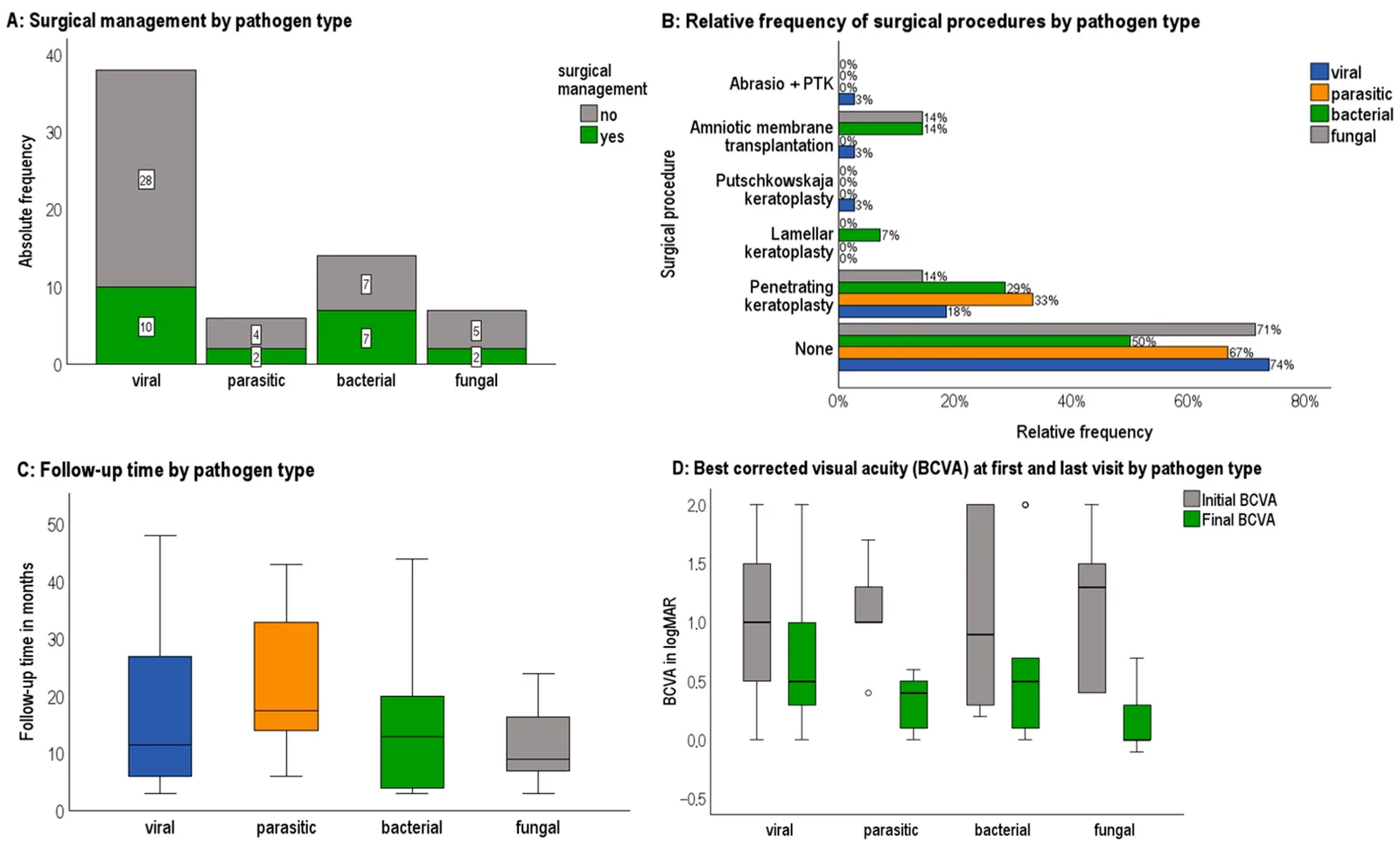
(**A**): Surgical management by pathogen type: Absolute frequency of patients requiring surgical intervention (green) versus medical treatment alone (gray). No statistically significant difference in surgical therapy was observed between the pathogen groups (*p* = 0.448, Fisher’s exact test). The total cohort consists of *n* = 65 (viral: *n* = 38, parasitic: *n* = 6, bacterial: *n* = 14, fungal: *n* = 7). (**B**): Realtive frequency of surgical procedures by pathogen type: Distribution of specific surgical techniques among the different pathogen groups. Percentages refer to the total number of patients within each pathogen group. A total of *n* = 21 surgical procedures were performed (viral: *n* = 10, parasitic: *n* = 2, bacterial: *n* = 7, fungal: *n* = 2). PTK = phototherapeutic keratectomy. (**C**): Follow-up time by pathogen type: Boxplots showing the distribution of follow-up duration in months for each group. (**D**): Best-corrected visual acuity (BCVA) at first and last visit by pathogen type: Comparison of initial BCVA (gray) and final BCVA (green) in logMAR. White circles indicate outliers.

Medical therapies are summarized in Table 3. Surgical frequency and procedures are presented in Figure 5A,B. Surgical rates did not differ significantly between groups (*p* = 0.448, Fisher’s exact test). In viral keratitis, surgery was most common in necrotizing keratitis (75.0%) and combined forms (36.4%).

Patients requiring surgery had significantly more severe initial findings (*p* = 0.019, Exact Fisher Test), but did not differ significantly in terms of age, pain, initial visual acuity or time between symptom onset and initial doctor’s visit.

Amniotic membrane transplantation (AMT) was performed in three patients (two with bacterial and one with fungal keratitis). The clinical rationales were distinct: In one bacterial case, AMT served a tectonic purpose to stabilize the cornea and lower the risk of perforation. In the remaining two cases (one bacterial, one fungal), the procedure was indicated to promote re-epithelialization of a persistent epithelial defect. Prior to surgery, the acute phase of infection had been successfully overcome by pathogen-specific therapy.

The mean time till surgical treatment was as follows:

- Fungal: 2.0 (±1.41) months (median 2.0 months).
- Bacterial: 4.86 (±5.3) months (median 6.0 months).
- Viral: 26.3 (±44.05) months (median 5.0 months).
- *Acanthamoeba*: 35.0 (±4.24) months (median 35.0 months).

#### 3.5.2. Topical Steroids

Steroid eye drops were administered to 27 patients (71.1%) with viral keratitis, two patients (33.3%) with *Acanthamoeba* keratitis, 12 patients (85.7%) with bacterial keratitis and four patients (57.1%) with fungal keratitis.

#### 3.5.3. Supportive Therapy

In addition to antimicrobial and surgical treatment, supportive therapy was administered to promote corneal healing. A total of 13 patients (20.0%) received autologous serum eye drops. Among these, the autologous serum was initiated during the acute phase of the infection in four cases (6.1%). Conventional lubricants were administered to 32 patients (49.2%) primarily during the regenerative phase.

#### 3.5.4. Antiviral Prophylaxis

Fifty percent of patients with viral keratitis were given oral acyclovir at prophylactic dosage. Prophylaxis was omitted in epithelial cases and in one patient with severe renal insufficiency. One patient discontinued prophylaxis due to lack of compliance.

### 3.6. Follow-Up

The mean follow-up time was 17.4 (±14.1) months for viral keratitis, 21.8 (±13.6) months for parasitic keratitis, 14.6 (±11.6) months for bacterial keratitis and 11.9 (±7.9) months for fungal keratitis [Figure 5C].

### 3.7. Visual Acuity and Outcome

The best-corrected visual acuity (BCVA) at initial presentation was as follows: viral 0.95 (±0.61) logMAR; *Acanthamoeba* 1.18 (±0.5) logMAR; bacterial 1.06 (±0.77) logMAR; and mycotic 1.07 (±0.66) logMAR.

There were no statistically significant differences in terms of age, pain or severity.

In cases of early consultation of bacterial keratitis (≤30 days), visual acuity was poorer (1.42 ± 0.66 logMAR) than in cases of late consultations (>30 days) (0.40 ± 0.35 logMAR) (*p* = 0.036, Mann–Whitney U test).

After medical therapy the BCVA increased in all pathogen groups:

- Viral from 0.85 (±0.57) to 0.55 (±0.51) logMAR.
- *Acanthamoeba* from 1.43 (±0.34) to 0.33 (±0.31) logMAR.
- Bacterial from 0.77 (±0.74) to 0.29 (±0.29) logMAR.
- Fungal from 0.94 (±0.76) to 0.10 (±0.23) logMAR.

Increases in visual acuity were significant for viral (*p* = 0.028, Wilcoxon test) and fungal (*p* = 0.042, Wilcoxon test) keratitis.

An increase in the BCVA was observed in all pathogen groups after surgical treatment as well:

- Viral from 1.22 (±0.67) to 1.07 (±0.69) logMAR.
- *Acanthamoeba* from 0.70 (±0.42) to 0.30 (±0.28) logMAR.
- Bacterial from 1.34 (±0.74) to 1.10 (±0.87) logMAR.
- Fungal from 1.40 (±0.14) to 0.35 (±0.49) logMAR.

However, these changes were not statistically significant.

For recurrence rates and complications, see Table 2. The recurrence rate was the same in viral keratitis patients with and without antiviral prophylaxis (50.0% each). Most recurrences occurred in combined keratitis (72.7%), followed by keratouveitis (63.6%) and necrotizing keratitis (50.0%). To the best of our knowledge, no recurrences occurred in patients with epithelial viral keratitis.

## 4. Discussion

### 4.1. Epidemiology and Risk Factors

#### 4.1.1. Viral Keratitis

In Germany, women have a higher life expectancy than men [17]. Since the risk of reinfection increases with age, women are more frequently affected by viral keratitis. Surgical procedures damage the corneal nerve plexus, and subsequent steroid administration reduces immune defense, making HSV reactivation more likely [18]. Notably, many of our patients had diabetes mellitus, compared to only 5% in the study of Rangel et al. [19]. Cardiovascular diseases are generally more prevalent in older patients; therefore, the etiological connection remains uncertain. In cases with an unclear risk profile, the herpetic origin should always be considered.

#### 4.1.2. *Acanthamoeba* Keratitis

The mean age of our cohort is higher than the 31–41 years reported in previous studies [20,21,22,23,24,25]. Consistent with publications from the German *Acanthamoeba* Registry, Tunisia, Australia, and Egypt, more women than men are affected [20,21,24,25]. Contact lens wear was the primary risk factor in our study, though others have reported trauma as more significant [23]. In general, the risk profiles vary by region:

- Low socioeconomic status: Male gender, young age, ocular trauma, and occupational exposure to contaminated water and organic materials.
- High socioeconomic status: Female gender because of higher rates of contact lens use [26,27].

#### 4.1.3. Bacterial Keratitis

Mean age for bacterial keratitis patients ranges from 49.8 to 57.6 years [28,29,30]. While the literature usually reports an equal gender distribution, men comprised 57.1% of our cohort [28,29,31]. Key risk factors include: contact lens use (primarily in younger patients) [30], OSD, ocular surgery and trauma [8,10,14]. In our cohort, only one patient wore contact lenses, possibly due to the small number of cases (14 patients total). In contrast, significantly more patients had OSD and had undergone previous surgical procedures.

#### 4.1.4. Fungal Keratitis

Fungal keratitis mostly affects young patients, with the mean age in the literature ranges from 44.9 to 60.4 years [32,33,34,35,36]. While the proportion of women in this study is low (14.3%), other publications report significant higher rates between 35.3% and 44.4% [32,34,35,36]. In France, a predominance of women was observed from 2014 to 2018 [33].

The etiology of fungal keratitis is closely linked to climate and risk factors. Candida infections primarily occur in temperate climates, particularly in patients with OSD, a history of ocular surgery or ongoing topical steroid use. In tropical/subtropical regions, filamentous fungi are the most common causative agents, with fungal keratitis often resulting from ocular trauma involving organic material. Typically male-dominated occupations in agriculture and crafts increase the risk for fungal infection [4]. However, in the Rostock region, the leading risk factors were contact lens use (42.9%) and previous ocular surgery (28.6%). The rising popularity of contact lens wear has led to a corresponding increase in fungal keratitis among female patients.

### 4.2. Symptoms and Clinical Findings

#### 4.2.1. Viral Keratitis

In accordance with Rangel et al., stromal keratitis was the most common finding [19]. However, a study from Milan also reported a higher frequency of keratouveitis accounting for approximately one-third of cases [37]. Our cohort’s relatively low rate of uncomplicated epithelial keratitis likely reflects our status as a tertiary care facility, where more complex cases are managed. Furthermore, 15 of the 38 patients had received prior treatment, which may have influenced our clinical findings.

#### 4.2.2. *Acanthamoeba* Keratitis

While the literature reports pain in 67% to 100% of *Acanthamoeba* keratitis cases, 83.3% of our patients experienced this symptom [20,22,23,24,25]. Pain is not typically an initial symptom, as perineuritis develops during the course of the infection. Consistent with previous studies, we found no correlation between clinical findings and pain perception [5,27].

The main clinical findings include ring-shaped stromal infiltrates, pseudodendritic epitheliopathy, perineuritis and anterior uveitis [5,12,20]. In our study, stromal infiltrate was present in all cases (100%). However, the classic ring-shaped configuration is not obligatory. It was observed in 50.0% of our patients, compared to 4–53% in other publications [20,21,22,23,24,25]. This variability underscores the need for advanced diagnostics especially in atypical cases. Additionally co-infections with other pathogens must be considered and treated, since *Acanthamoeba* feed on bacteria and fungi [20].

#### 4.2.3. Bacterial Keratitis

Our patient group presented with the typical symptoms described in the literature including pain, epiphora, and reduced visual acuity. The clinical findings were consistent with the standard manifestation of bacterial keratitis [7,9,38].

#### 4.2.4. Fungal Keratitis

Fungal keratitis typically presents as white, feathery, raised infiltrates with an overlying epithelial defect. Although satellite lesions are a classic sign, they are not pathognomonic and can also occur in bacterial or *Acanthamoeba* keratitis [4]. Consistent with other reports, the most common findings in our cohort are infiltrates and epithelial defects that extend to deep ulcers [32,33,34,35].

Hypopyon occurred in 42.9% of fungal keratitis, which sits at the upper end of the 17–42.1% range reported in the literature [33,34,35]. Therefore, the presence of hypopyon should always prompt the consideration of fungal infection alongside bacterial and parasitic causes.

### 4.3. Diagnostics

Patients with viral, bacterial and *Acanthamoeba* keratitis took longer to seek medical care than patients with fungal keratitis [Figure 4A]. Severeal factors may contribute to this delay. First, the intial point of medical contact was defined as specialized ophthalmological care. A portion of the cohort received prior treatment from general practitioners. They only sought specialist consultation after the intial therapy failed. Second, the study was conducted in a predominantly rural region, where geographical distances to specialized centers may influence healthcare-seeking behavior. Lastly, the data is based on patient history and therefore subject to recall bias. Early, non-specific symptoms, such as mild discomfort or light sensitivity, might have been reported by patients as the onset of infection, even if a direct causal link cannot be definitely established.

#### 4.3.1. Viral Keratitis

The majority of patients were diagnosed clinically, with viral DNA detected via PCR in only one-third of cases. This rate is consistent with findings by Miserocchi et al. [37]. This low detection rate is largely due to the limitations of corneal sampling. Stromal herpetic keratitis cannot be diagnosed with certainty using PCR, because of its immunological genesis and the intact epithelial barrier. Furthermore, due to the usually small sample size, not every attempt at detection is successful [10].

#### 4.3.2. *Acanthamoeba* Keratitis

The mean time to consultation was consistent with the existing literature [20,21,22,23,24,25]. In our cohort, two-thirds of cases were diagnosed using confocal microscopy and one-third clinically. Neither PCR nor cultural detection was successful. In the German *Acanthamoeba* Registry, confocal microscopy played a minor role compared to histology [27]. Confocal microscopy is a noninvasive diagnostic tool but requires an experienced examiner. Despite its advantages, confocal microscopy is neither available nor routinely performed in every eye clinic. However, given the limitations of PCR and culture, combining multiple diagnostic procedures is essential to enable rapid, pathogen-specific therapy [39].

#### 4.3.3. Bacterial Keratitis

We hypothesize that the significantly lower BCVA in bacterial keratitis patients caused greater distress, leading to an earlier specialist consultation. Unfortunately, the literature currently lacks data to support this specific correlation.

Cultural pathogen detection should always be attempted in cases of suspected bacterial keratitis with atypical clinical findings to rule out other causative pathogens [13]. As resistance increases, antibiograms will play an increasingly important role in monitoring antibiotic therapy [7,40].

#### 4.3.4. Fungal Keratitis

The rapid consultation time for our fungal keratitis patients cannot be attributed to increased clinical severity, as findings were similar to other cohorts [33,34,35,36]. The proximity and availability of medical care may have played a role. However, there is no data available supporting this thesis.

Bakken et al. recommend IVCM as a supplementary diagnostic tool due to its rapid availability and noninvasive nature. However, due to its low sensitivity, it cannot be used as a stand-alone diagnostic procedure [41]. Confocal microscopy can be very helpful when other methods, such as culture or PCR, fail to yield results.

### 4.4. Management

#### 4.4.1. Viral Keratitis

While various antiviral substances show similar efficacy, acyclovir remains the preferred treatment due to its high selectiveness as a prodrug [10]. Our surgical intervention rates were notably lower than the pKP rate of 35% reported by Rangel et al. Their study also demonstrated superior outcomes for elective procedures compared to keratoplasty à chaud [19]. Recent findings by Yang et al. further support the success of medical management in severe cases [42]. Consequently, a “medical-first” approach, potentially supplemented by minor procedures to stabilize the cornea, such as amniotic membrane transplantation, should be prioritized.

#### 4.4.2. *Acanthamoeba* Keratitis

The patients in our study and in the *Acanthamoeba* Registry most commonly received a triple combination of topical PHMB, propamidine and antibiotics [20]. In Egypt and Austria, a combination of PHMB and Brolene is more common [22,25]. Our surgical rate of 33.3% aligns with the 7.7–40.4% range reported in the literature, reflecting the often recalcitrant nature of parasitic infection [20,21,22,23,25].

#### 4.4.3. Bacterial Keratitis

Although various topical antibiotics are equally effective, fluoroquinolones are generally better tolerated [43]. However, the global rise in resistance to fluoroquinolones presents ophthalmologists with new challenges in the management of bacterial keratitis [7,40]. Regarding topical steroid therapy, we observed no significant increase in visual acuity, mirroring the findings of Srinivasan et al. While complication rates were comparable, the literature suggests that specific subgroups—such as Nocardia infections or patients receiving steroids within the first 48 h of infection—do not benefit from steroid administration [44].

#### 4.4.4. Fungal Keratitis

Olivier et al. showed a decline in keratoplasty between 1993 and 2008, and 2014 and 2018. Our patients presented after 2010, mostly within 3 (±3) days of symptom onset. Early diagnosis and initiation of therapy likely contributed to our lower surgical rate [4].

Treatment regimens varied, ranging from antifungal monotherapies to triple combinations of antifungals and antibiotics. The rate of patients undergoing surgery in the literature was slightly higher than in our cohort, at 11–42.2% for keratoplasty [32,33,35,36].

#### 4.4.5. Supportive Therapy

The potential of autologous serum eye drops to reduce the need for surgical treatment was evaluated. In our cohort, 13 patients (20.0%) received autologous serum. Notably, only four of these patients (6.1% of the total cohort) received this supportive therapy during the acute phase of infection. The majority of supportive care consisted of conventional lubricants (*n* = 32, 49.2%), which were typically introduced in the later stages to promote corneal regeneration. Therefore, the limited and often delayed use of autologous serum is unlikely to have significantly altered the surgical rates in this study.

### 4.5. Visual Acuity and Outcome

Our results suggest that medical and surgical treatment approaches for infectious keratitis yield equivalent final visual outcomes, as both lead to comparable increases in visual acuity. Daas et al. also found no difference in efficacy between the two forms of therapy for *Acanthamoeba* keratitis [20]. Like Rangel et al., we observed most surgical interventions in necrotizing herpes keratitis, which is associated with the poorest visual outcome [19].

Several factors influenced the visual outcomes in our cohort.

- Lens status: Only five patients (viral three, *Acanthamoeba* two) underwent cataract surgery during the follow-up period. However, given the advanced age of the viral keratitis group, pre-existing lens opacities may have limited the visual recovery. Subsequent cataract surgery could further improve BCVA.
- Early consultation: Patients with bacterial keratitis who presented after 30 days exhibited significantly better visual acuity. While this appears counterintuitive given the progressive nature of the disease, it likely reflects a selection bias. Patients with peripheral lesions and minimal visual impairment may delay seeking medical care, whereas those with central involvement and acute vision loss present earlier.
- Recurrence rates: The recurrence rate deeply impacts visual acuity and outcome in cases of viral keratitis [45]. Although recurrence rates can be reduced by antiviral prophylaxis, our cohort showed a high recurrence rate of 50.0% regardless of prophylaxis [11]. This is notably lower than the reported 65.1% in an Italian study (where 71.4% received prophylaxis), but still remains a clinical challenge [37]. The high recurrence rate in bacterial and mycotic keratitis may have been influenced by our inclusion criteria. Patients without recurrence were less likely to complete the required three-month follow-up.

### 4.6. Limitations

Our study has several limitations. First, its retrospective design inherently leads to incomplete datasets and potential variations in diagnostic and therapeutic protocols. However, all patients were examined and treated according to the specialist standard of care under the supervision of senior consultants. Second, while the study aimed to characterize infectious keratitis at a tertiary referral center, our data may not be representative of all cases in the Rostock area. Patients with uncomplicated courses are often managed solely by outpatient ophthalmologists. Furthermore, because our center serves a predominantly rural region, the findings regarding healthcare-seeking behavior and referral times may differ from those in urban settings. Lastly, some data are based on patient history and are subject to recall bias.

## 5. Conclusions

Infectious keratitis remains a complex clinical challenge, with significant variations in epidemiology, clinical presentation, and management depending on the underlying pathogen. Our analysis identifies distinct risk factors influenced by regional characteristics, with contact lens wear and prior ocular surgery playing a predominant role in the Rostock cohort.

A key finding of this study is the statistical difference in pain perception across pathogen groups (*p* = 0.009), which underscores the clinical importance of symptomatic profiles in differential diagnosis. Because pathognomonic clinical features are not always present, clinical assessment should be complemented by a multimodal diagnostic approach. While PCR, staining and culture remain essential, in vivo confocal microscopy proved to be an invaluable noninvasive tool, bridging the gap in culture-negative cases.

Although visual outcomes were comparable between medical and surgical management, surgical intervention should be reserved for severe or refractory cases. Recurrence in viral keratitis remains a significant factor influencing prognosis. Our findings underscore the importance of early diagnosis, pathogen-specific therapy, and awareness of regional risk factors to optimize the clinical course.

## Figures and Tables

**Table 1:** Additional clinical features to further assess the severity of infectious keratitis.

Severity	Clinical Features
Light	Small, subepithelial or perineural infiltrates, keratitis superficialis punctata, epithelial opacity, fluorescein-positive lesions, reduced corneal sensitivity
Moderate	Focal stromal opacity and edema, diffuse and bigger infiltrates, small ulcus, neovascularization of the cornea, hypopyon, anterior chamber cells, Tyndall+, extinguished corneal sensitivity
Severe	Diffuse and deep infiltrates, geographical and deep ulcera, complete corneal opacity, pannus, descemetocele, scarring, hyphema, anterior synechiae, scleritis

**Table 2:** Risk factors, clinical findings and complications of infectious keratitis.

	Viral	Parasitic	Bacterial	Fungal
Characteristics				
Mean age in years ^1^	64 (±15)	53 (±17)	57 (±24)	45 (±20)
Male ^2^	16/38 (42.1%)	2/6 (33.3%)	8/14 (57.1%)	6/7 (85.7%)
Female ^2^	22/38 (57.9%)	4/6 (66.7%)	6/14 (42.9%)	1/7 (14.3%)
Risk factors				
Previous surgical procedure	7/38 (18.4%)	0/6 (0%)	2/14 (14.3%)	2/7 (28.6%)
Trauma	2/38 (5.3%)	0/6 (0%)	1/14 (7.1%)	2/7 (28.6%)
Contact lens wear	0/38 (0%)	5/6 (83.3%)	1/14 (7.1%)	3/7 (42.9%)
Ocular surface disease	10/38 (26.3%)	0/6 (0%)	5/14 (35.7%)	0/7 (0%)
Diabetes mellitus	12/38 (31.6%)	0/6 (0%)	1/14 (7.1%)	0/7 (0%)
Lens status ^3^				
Phakic	23/38 (60.5%)	5/6 (83.3%)	13/14 (92.9%)	7/7 (100%)
Pseudophakic	13/38 (34.2%)	0/6 (0%)	1/14 (7.1%)	0/7 (0%)
Aphakic	1/38 (2.6%)	1/6 (16.7%)	0/14 (0%)	0/7 (0%)
Clinical findings				
Ulcer	7/38 (18.4%)	2/6 (33.3%)	6/14 (42.9%)	3/7 (42.9%)
Epithelial defect	28/38 ^4^ (73.7%)	2/6 (33.3%)	7/14 (50.0%)	2/7 (28.6%)
Infiltrate	13/38 (34.2%)	6/6 ^5^ (100%)	5/14 (35.7%)	7/7 (100%)
Edema	13/38 (34.2%)	2/6 (33.3%)	5/14 (35.7%)	3/7 (42.9%)
Descemet folds	13/38 (34.2%)	3/6 (50.0%)	3/14 (21.4%)	4/7 (57.1%)
Reduced corneal sensitivity	22/38 (57.9%)	3/6 (50.0%)	4/14 (28.6%)	0/7 (0%)
Uveitis anterior	17/38 (44.7%)	1/6 (16.7%)	5/14 (35.7%)	3/7 (42.9%)
Hypopyon	0/38 (0%)	1/6 (16.7%)	2/14 (14.3%)	3/7 (42.9%)
Management				
Inpatient ^6^	23/38 (60.5%)	3/6 (50.0%)	9/14 (64.3%)	6/7 (85.7%)
Outpatient ^6^	15/38 (39.5%)	3/6 (50.0%)	5/14 (35.7%)	1/7 (14.3%)
Complications				
Scarring	18/38 (47.4%)	3/6 (50.0%)	2/14 (14.3%)	4/7 (57.1%)
Vascularization	18/38 (47.4%)	1/6 (16.7%)	6/14 (42.9%)	4/7 (57.1%)
Corneal dent/thinning	2/38 (5.3%)	0/6 (0%)	3/14 (21.4%)	0/7 (0%)
Perforation	4/38 (10.6%)	0/6 (0%)	2/14 (14.3%)	0/7 (0%)
Secondary glaucoma	11/38 (28.9%)	1/6 (16.7%)	0/14 (0%)	0/7 (0%)
Enucleation	2/38 (5.3%)	1/6 (16.7%)	0/14 (0%)	0/7 (0%)
Recurrence	19/38 (50.0%)	2/6 (33.3%)	7/14 (50.0%)	2/7 (28.6%)

Cumulative percentage > 100% possible, because of multiple risk factors, multiple clinical findings and multiple complications. ^1^ No statistically significant difference in age was observed between the pathogen groups (*p* = 0.069, Kruskal–Wallis test). ^2^ No statistically significant difference in gender distribution was observed between the pathogen groups (*p* = 0.140, Fisher’s exact test). ^3^ Lens status at the time of the initial presentation. ^4^ 8/28 (28.6%) with dendritic formation. ^5^ 3/6 (50.0%) with ring formation. ^6^ No statistically significant difference in case management was observed between the pathogen groups (*p* = 0.612, Fisher’s exact test).

**Table 3:** Medical therapy for infectious keratitis.

Treatment Regimen	Dosage	Absolute (Relative) Frequency
Viral		
Fluroquinolone	5× daily	1/38 (2.6%)
Acyclovir (Ganciclovir) ointment	5× daily	10/38 (26.3%)
Acyclovir 400–800 mg p.o.	5× daily	3/38 (7.9%)
Acyclovir ointment + Acyclovir 400–800 mg p.o.	5× daily	24/38 (63.2%)
Addition of topical antibiotics ^1^	5× daily to q1h	10/38 (26.3%)
*Acanthamoeba*		
PHMB + Propamidine	q1h to q1/2h	3/6 (50.0%)
Propamidine	q1h to q1/2h	3/6 (50.0%)
Addition of topical antibiotics ^1^	q1h	5/6 (83.3%)
Bacterial		
Macrolide	5× daily	1/14 (7.1%)
Aminoglycoside	5× daily to q1h	2/14 (14.3%)
Fluroquinolone	5× daily to q1/2h	3/14 (21.4%)
Aminoglycoside + Fluroquinolone	q1h to q1/2h	7/14 (50.0%)
Neomycin sulfate + Polymyxin B sulfate + Bacitracin	5× daily	1/14 (7.1%)
Fungal		
Fluconazole	q1h	2/7 (28.8%)
Voriconazole 2%	q1h	3/7 (42.9%)
Voriconazole 2% + Amphotericin B 0.25%	q1h	2/7 (28.8%)
Addition of topical antibiotics ^1^	5× daily to q1h	4/7 (57.1%)

The treatment regimens and dosages specified were intitiated during the acute phase of infection. Dosages were subsequently tapered based on the individual clinical response. ^1^ The most frequently used topical antibiotics were fluroquinolones and aminoglycosides.

## Data Availability

The datasets presented in this article are not readily available because of privacy restrictions and ethical considerations. Requests to access the datasets should be directed to thomas.fuchsluger@med.uni-rostock.de.

## Abbreviations

The following abbreviations are used in this manuscript:

WHO	World Health Organization
OSD	Ocular surface disease
IVCM	In vivo confocal microscopy
PCR	Polymerase chain reaction
pKP	Penetrating keratoplasty
BCVA	Best-corrected visual acuity
PHMB	Polyhexamethylene biguanide
HSV	Herpes simplex virus
